# Editorial: Ferroptosis in stroke, neurotrauma and neurodegeneration, volume II

**DOI:** 10.3389/fncel.2023.1238425

**Published:** 2023-07-05

**Authors:** Weilin Xu, Zhen-Ni Guo, Anwen Shao

**Affiliations:** ^1^Department of Neurosurgery, The Second Affiliated Hospital, Zhejiang University School of Medicine, Hangzhou, Zhejiang, China; ^2^Key Laboratory of Precise Treatment and Clinical Translational Research of Neurological Diseases, Hangzhou, Zhejiang, China; ^3^Department of Neurology, The First Hospital of Jilin University, Changchun, China

**Keywords:** ferroptosis, stroke, neurotrauma, neurodegeneration, cell death

Ferroptosis, a new form of cell death, is characterized by the accumulation of intracellular iron and lipid reactive oxygen species (ROS) (Deng et al., 2023). The primary morphologic manifestations of ferroptosis include cell volume shrinkage and increased mitochondrial membrane density. This process of ferroptosis differs from other apoptotic types of cell death such as necroptosis and pyroptosis (Costa et al., 2023). Growing evidence indicates that ferroptosis plays key roles in neurological diseases such as stroke, traumatic brain injury (TBI), and neurodegenerative diseases [Alzheimer's disease (AD), Parkinson's disease (PD), amyotrophic lateral sclerosis (ALS), vascular dementia (VD), Huntington's disease (HD)] (Costa et al., 2023; Du et al., 2023). Ferroptosis inhibition has been shown to protect neurons and ameliorate cognitive impairment in various disease animal models (Li et al., 2022; Zhang et al., 2023). However, to date, the underlying mechanisms that how ferroptosis causes neurological injuries are still unclear. This Research Topic finally published nine studies, which extensively explored the relationships between ferroptosis and neurological diseases (stroke, TBI, and neurodegenerative diseases), including underlying mechanisms of ferroptosis, potential targeting, and the relationship between ferroptosis and other kinds of cell death (necroptosis and pyroptosis).

## Ferroptosis and stroke

Zhu et al. adopted the method of bioinformatics analysis to explore the ferroptosis-related ceRNA regulation network in intracranial aneurysm. They analyzed data from the Gene Expression Omnibus (GEO) datasets and tried to identify differentially expressed genes (DEGs), differentially expressed miRNAs (DEMs), and differentially expressed lncRNAs (DELs) in intracranial aneurysm. In all, 30 ferroptosis DEGs, five key DEMs, and 17 key DELs were screened and they found that CeRNA (PVT1-hsa-miR-4644-SLC39A14 and DUXAP8-hsa-miR-378e/378f-SLC2A3) overexpression networks were associated with ferroptosis in intracranial aneurysm. Pan et al. extensively elucidated the underlying mechanisms of ferroptosis in hemorrhagic stroke. They reported that ferroptosis plays important roles in the hemorrhagic stroke. The mechanisms include ion overloaded, lipid peroxidation, and dysfunctional antioxidant system. Moreover, they also discussed the crosstalk between ferroptosis and other types of neuronal death or autophagy. Finally, they pointed out that ferroptosis marker detection, diversity of ferroptotic cell, and the uncertainty about the magnitude and duration of ferroptotic action are mainly the research dilemma and will be the research prospects of ferroptosis. The third study by Cao et al. also introduced the underlying mechanism and crosstalk of ferroptosis with other modes of neuronal death after intracerebral hemorrhage. Except for the overlaping content showed by Pan et al., Cao et al. discussed several therapeutic targets of inhibitors of ferroptosis in ICH, including selenium supplementation, iron chelators (DFX, VK-28, deferiprone), lipoxygenase inhibitors, and DMT1 inhibitor (Ebselen). The study by Chen et al. extensively introduced different patterns of neuronal death and their molecular mechanisms after subarachnoid hemorrhage (SAH), including necrosis, apoptosis, pyroptosis, autophagy, necroptosis, and ferroptosis. In ferroptosis, they emphatically introduced three primary anti-ferroptosis systems, GPX4–GSH–cysteine system, FSP1–CoQ10–NAD (P)H system and GCH1-BH4-DHFR system. These studies extensively demonstrated the underlying mechanisms of ferroptosis in stroke and its potential therapeutic targets, which provides key information for future study of ferroptosis in stroke.

## Ferroptosis and neurodegenerative diseases

Among the rest, three studies emphasized the important roles of ferroptosis in neurodegenerative diseases. Wang et al. applied bioinformatic analysis to explore ferroptosis-related biomarkers for AD. They also analyzed the data from GEO datasets and performed enrichment analyses of protein–protein interaction (PPI), the Gene Ontology (GO), and Kyoto Encyclopedia of Genes and Genomes (KEGG) pathway. Twenty-four ferroptosis-related genes were identified as target genes. Finally, ASNS and SENS2 are screened as potential diagnostic biomarkers for AD and provide additional evidence regarding the essential role of ferroptosis in AD. In another study, Fu et al. focused on investigating the expression pattern of ferroptosis and iron metabolism-related genes (FIRGs) in amyotrophic lateral sclerosis (ALS). They performed a conjoint analysis of bulk-RNA sequence and single-nucleus RNA sequence data using the datasets from GEO. Fifteen FIRGs was identified as target genes. Final analysis of bulk single-nucleus RNA-seq data showed that CHMP5 was expressed significantly higher in ALS than pathologically normal (PN), specifically in excitatory neuron populations. The third study comprehensively reviewed the relationship of ferroptosis and neurodegenerative diseases. Firstly, they introduced the mechanisms of ferroptosis, including transport and storage of iron in the brain, the glutamate/cysteine antiporter in ferroptosis and lipid peroxidation in ferroptosis. In the second part, they reviewed the roles of ferroptosis in each neurodegenerative disease, such as AD, vascular dementia (VD), PD, HD, ALS, and TBI. These three studies interestingly showed the molecular patterns and underlying mechanisms of ferroptosis in neurodegenerative diseases.

## Ferroptosis and other neurological diseases

The remaining two studies provided another view of neuronal death in neurological diseases. Li et al. used bioinformatic analysis to identify cuproptosis-related genes (CRGs) on disease progression and the immune microenvironment in acute spinal cord injury (ASCI) patients. Their results showed that dihydrolipoamide dehydrogenase (DLD) affects the ASCI immune microenvironment by promoting copper toxicity, leading to increased peripheral M2 macrophage polarization and systemic immunosuppression. In the second study, Shi et al. demonstrated the underlying mechanisms of deep brain stimulation (DBS) for treatment of refractory obsessive-compulsive disorder (OCD).

In conclusion, ferroptosis plays important roles in various neurological diseases and its pathological mechanism is complicated. Ferroptosis differs from other apoptotic types of cell death (necrosis, apoptosis, pyroptosis, autophagy, necroptosis, and ferroptosis), however, it also connects with many physiological processes (apoptosis, autophagy, oxidative stress, inflammation). This Research Topic comprehensively discussed the underlying mechanisms and potential targets of ferroptosis in each neurological disease, which provides key insights for further study of ferroptosis in the central nervous system.

## Author contributions

All authors listed have made a substantial, direct, and intellectual contribution to the work and approved it for publication.
